# Prevalence and risk factors for subclinical atherosclerosis amongst adults living with HIV in University of Abuja Teaching Hospital, Gwagwalada

**DOI:** 10.3389/frph.2023.1092211

**Published:** 2023-02-03

**Authors:** Taiwo A. Adedokun, Vivian G. Kwaghe, Oluwasanmi Adedokun, Titilope Badru, Augustine N. Odili, Jacob Alfa, Hadijat O. Kolade-Yunusa, Dike B. Ojji

**Affiliations:** ^1^ Department of Internal Medicine, University of Abuja Teaching Hospital, Gwagwalada, FCT, Nigeria; ^2^ Center for International Health, Education, and Biosecurity (Ciheb), Maryland Global Initiatives Corporation (MGIC) - an Affiliate of University of Maryland Baltimore, Abuja, Nigeria; ^3^ Strategic Information Department, FHI360, Abuja, Nigeria

**Keywords:** ART, CIMT (Carotid intima-media thickness), CVD (cardiovascular disease), HIV - human immunodeficiency virus, sub-clinical atherosclerosis

## Abstract

**Background:**

Subclinical atherosclerosis characterizes cardiovascular diseases (CVD), and Human Immunodeficiency Virus (HIV) infection and antiretroviral therapy (ART) are identified risk factors for atherosclerosis. Meanwhile, data on HIV and atherosclerosis in Nigeria are limited.

**Objectives:**

We sought to estimate the prevalence of subclinical atherosclerosis and associated risk factors amongst adult persons living with HIV/AIDS (PLHIV) enrolled at University of Abuja Teaching Hospital, Gwagwalada, Abuja (UATH).

**Methods:**

This was a cross-sectional study of 277 consecutively selected PLHIV ≥18 years enrolled for HIV care and treatment at UATH. Pretested structured questionnaire was used to collect data from consenting ART-experienced and ART-naïve patients on risk factors of atherosclerosis. Carotid intima media thickness (CIMT) ≥0.71 mm as measured by Doppler ultrasonography was used to identify patients with sub-clinical atherosclerosis. Two logistic regression models with (Model-A) and without (Model-B) traditional risk factors were fitted to identify risk factors of subclinical atherosclerosis.

**Results:**

Participants' mean age was 39.44 ± 10.71 years with female preponderance (64.26%). Overall prevalence of subclinical atherosclerosis was 43.32% (62.25% in ART-experienced). Model-A identified male sex [AOR 4.33(1.74–10.76), *p* = 0.002], advancing age [30–39 years AOR 5.95(1.31–26.96), *p* = 0.021]; ≥40 years AOR 19.51(4.30–88.56), *p* ≤ 0.001), advancing HIV infection [≥WHO stage II AOR 4.19(1.11–15.92), *p* = 0.035], hypercholesterolemia [AOR 3.88(1.47–10.25), *p* ≤ 0.001] and ≥5 year duration on ART [AOR 9.05(3.16–25.92), *p* ≤ 0.001] as risk factors of subclinical atherosclerosis. In Model-B (excluding traditional risk factors) on the other hand, advancing HIV infection [≥WHO stage II AOR 3.93(1.19–13.042), *p* = 0.025] and duration on ART [≥5 years AOR 11.43(4.62–28.29), *p* = 0.001] were found as risk factors of subclinical atherosclerosis.

**Conclusion:**

Subclinical atherosclerosis was higher in ART-experienced patients, and this was irrespective of presence or absence of traditional risk factors. And advancing HIV disease and duration on ART were found as significant risk factors for subclinical atherosclerosis. We therefore recommend routine CVD risk screening in PLHIV.

## Background

Human Immunodeficiency Virus (HIV) infected individuals appear to have significantly higher risk of myocardial infarction and coronary heart disease relative to HIV negative individuals (1–4). With the advent of lifelong antiretroviral treatment (ART), HIV positive persons have increased life expectancy (5, 6). This increasing life expectancy is subsequently associated with increased risk of cardiovascular diseases (CVD) as increasing age is an independent risk for CVD (7–9). In addition to the identified traditional risk factors of CVD, persons living with HIV have increased risk of atherosclerosis due to use of antiretroviral (ARV) drugs and inflammatory factors related to HIV infection (10–13). Several studies have found an association between use of ARV drugs especially protease inhibitors and the development of atherosclerosis (14–17). Protease inhibitors are specifically associated with dyslipidaemia, thus increasing the risk of atherosclerosis (18), which is the hallmark of CVD (19, 20). Atherosclerosis which is characterized by hardening of arteries through the accumulation of plaques presents clinically as cardiovascular disease (CVD) events such as stroke and myocardial infarction (20). Due to the increasing morbidity and mortality associated with CVD (4), it is desirable to detect subclinical signs of CVD early so as to institute prompt management and delay the manifestation of the overt disease.

Meanwhile, the presence of atherosclerosis can be diagnosed early at subclinical levels using carotid intima media thickness (CIMT) as a measure (21–23), and increases in CIMT has been shown to be predictive of future CVD events (19). Both common carotid intima media thickness (CCA IMT) and internal carotid artery intima media thickness (ICA IMT) have been used as a measure of subclinical atherosclerosis. Common carotid intima media thickness (CCA IMT) is however preferred because it is easier to measure and results are more reproducible (24–26).

A Ugandan study to estimate the prevalence of subclinical atherosclerosis among HIV infected adults using a CIMT of ≥ 0.78 mm as cut off found an 18% prevalence (27). However, other studies among PLHIV in high income countries using higher CIMT cut-off values of 0.80 mm and 0.90 mm found prevalence of 65% and 41.7% respectively (28, 29). A Nigerian study by Yunusa et al. (30), comparing CIMT in normotensives and hypertensives found a mean CIMT of 0.61 ± 0.10 mm in normotensive individuals. In our environment, there is limited literature regarding the prevalence of subclinical atherosclerosis in HIV positive persons and the interplay of traditional risk factors of CVD and HIV infection and treatment, thus necessitating this study. This study estimated the prevalence of subclinical atherosclerosis amongst PLHIV enrolled at University of Abuja Teaching Hospital, Gwagwalada and describes the associated risk factors amongst this population.

## Materials and methods

### Study design

We conducted a cross-sectional study amongst HIV infected adults 18 years and above at the University of Abuja Teaching Hospital, Gwagwalada (UATH). Our study population comprised both ART experienced patients with at least six months of ART as well as ART naïve patients who were newly enrolled into care.

### Study setting and context

UATH is a 350-bedded tertiary facility located in Gwagwalada in the Federal Capital Territory in Nigeria. UATH runs clinic for persons infected with HIV with about 4,864 patients currently receiving life-long ART as of December 2018 (3,832 on first-line regimen and 1,032 on second-line regimen), and an average monthly ART enrollment rate of about 30 patients per month.

HIV testing services at UATH are routinely provided at multiple service delivery points including the general outpatient department, inpatient wards, the main laboratory, antenatal clinic, labour ward, TB clinic, family planning clinic, and sexually transmitted infection clinic using serial algorithm based on the Nigerian guidelines (31). Patients testing positive are retested by another tester using the same algorithm before referral to the ART clinic for enrollment into care (32). Nigeria's HIV treatment guidelines currently adopts the WHO “test and treat” strategy, hence all HIV positive persons are considered eligible for ART irrespective of CD4 counts or WHO staging. Following enrollment, patients are commenced on adherence counselling and are expected to initiate treatment immediately or as soon as possible, preferably within two weeks of testing HIV positive. ART clinics are run by physicians daily at the UATH and patients on treatment are provided with one-month prescription for the first time, subsequently 3-monthly at each visit. Laboratory monitoring with viral load estimation is carried out after six months on ART and annually subsequently. Preferred first-line regimen for adults based on national ART guidelines are tenofovir + lamivudine + dolutegravir (TDF + 3TC + DTG). Alternate first-line options include tenofovir + lamivudine (or emtricitabine) + efavirenz (or nevirapine) (TDF + 3TC (or FTC) + EFV (or NVP)), or zidovudine + lamivudine + efavirenz (or nevirapine) [AZT + 3TC + EFV (or NVP)], or abacavir + lamivudine + dolutegravir (ABC + 3TC + DTG) all as fixed dose combinations (33), while the preferred second-line ART regimen are zidovudine or tenofovir + lamivudine + lopinavir or atazanavir (AZT or TDF + 3TC + LPV/r or ATV/r) (32).

### Sampling and sample size estimation

Using Kish and Leslie's formula for prevalence studies, and assuming a prevalence of 18% (27) for subclinical atherosclerosis, and a precision of 5% between the assumed (referenced) prevalence and our study estimates, at a 5% level of significance with a 10% correction for non-response, we estimated a minimum sample size of 252 for this study. Our sample distribution was 50% for ART naïve patients and 50% for ART experienced patients. ART experienced patients were recruited into the study using a proportional allocation of samples of 4 first-line to 1 second line based on the numbers currently on ART in the facility. Based on the sampling plan, participants sample distribution was expected to be 126 ART naïve HIV positive persons, 101 HIV positive persons on first-line regimen and 25 persons on second-line regimen. However, the number of patients on second-line ART were doubled to achieve a minimum sample size of 50 for regression analysis based on statistical rule of thumb (34) to have a more robust sample for analysis, making a total sample size of 277.

Eligible patients enrolled into HIV care and treatment who consented to take part in the study were consecutively enrolled into the study until the required sample size was achieved for each group. Persons with history of cardiovascular risk including hypertension, diabetes, peripheral vascular disorder, myocardial infarction, heart failure, or stroke, who are likely to have clinical evidence of atherosclerosis were excluded from the study. Other exclusion criteria include persons with life-threatening opportunistic infection requiring critical care, or cognitive impairment precluding the patient from providing appropriate responses to interview questions or providing consent.

### Data collection and outcome measures

A structured data collection tool divided into four sections was used for data collection. These sections are: (1) screening questions to rule out study exclusion criteria, (2) sociodemographic and HIV related clinical characteristics of participants, (3) physical examination findings, and (4) laboratory and radiology results. The data collection tool was pretested and validated with 10 patients at UATH preceding the study. Pretest data were excluded from the analysis.

The primary outcome measure for this study was the presence of subclinical atherosclerosis amongst PLHIV enrolled in this study. Subclinical atherosclerosis was defined as carotid intima media thickness ≥0.71 mm as measured by Doppler ultrasonography (30). Informed consent was obtained from eligible participants identified and study identification number assigned. Survey questions were administered, and physical examinations were carried out, following which referrals were made for radiological and laboratory investigations. ART naïve patients were recruited into the study at the ART clinic at the time of enrollment into care just before they commenced ART, while ART experienced patients were recruited when they visited the facility for their routine clinical check-up and drug refills.

### Physical examination

Physical examination was carried out to check for signs and symptoms of opportunistic infections with classification of participant into applicable WHO clinical stages as elucidated in the WHO clinical staging criteria (35, 36). Blood pressure measurement was carried out in accordance with European Society of Cardiology (ESC) guideline recommendations (37) using a mercury sphygmomanometer (Accoson, England). The mean of the last two of three consecutive blood pressure measurements at 2-minutes intervals was used for the analysis. Participants' were weighed using a Seca digital weighing scale to the nearest 0.5 kilograms (38). Height was measured to the nearest 0.5 meters. The waist/hip ratio was estimated for each participant by measuring both the waist and hip circumference in centimeters and taking the ratio. The waist circumference was obtained by placing the measuring tape midway between the uppermost border of the iliac crest and the lower margin of the costal margin (39). The hip circumference was obtained by placing the tape parallel to the floor and around the widest portion of the buttocks.

### Laboratory/radiological procedures

Carotid Doppler ultrasonography was performed using a high resolution 10 MHz linear array transducer of LOGIC F series GE ultrasound machine (© 2016, General Electric Company, UK) by a trained cardiologist. Participants were requested to remove jewelry around the neck. Ultrasound of the carotid intima media was performed with the subject lying supine on the couch to the right of the examiner with pillow support under the neck to achieve the desired neck extension and head turned 45° away from the side being scanned. Adequate amount of coupling gel was applied to the scan area to eliminate air gap between probe and skin surface. Right and left CCA were located by longitudinal and transverse scans. Three measurements of the CIMT were obtained at the far wall on each side, each at 1 cm proximal to the right and left carotid bulb. The mean of three point measurements each of CIMT thickness for both right and left CCA were calculated and used for the analysis.

Laboratory request forms carrying the participant's study identification number were given to the patient and requested to return the following day for laboratory tests after an overnight fast of 8 to 12 h. On return, about 10 mls of venous blood sample was obtained under aseptic conditions. One drop of blood was used to test for fasting blood sugar using an Accucheck glucometer, while the rest of the sample was divided in aliquots of about 5 mls into vacutainer tube containing ethylene diamine tetra-acetic acid (EDTA) anticoagulant by Becton Dickinson, and plain bottle, for viral load testing and lipid profile, respectively. The blood was centrifuged for 10 min at 3,000 rpm within 2–4 h of collection, and the separated plasma and serum respectively were stored at −20°C. Lipid profile analysis was done using Landwind C100 Plus Chemistry Autoanalyzer for the total cholesterol (TC), High density lipoprotein-cholesterol (HDL-C), and triglycerides (TG). Low density lipoprotein-cholesterol (LDL-C) was calculated from TC, HDL-C and TG using Friedewald formulae (LDL-C = TC-TG/ 5 + HDL-C) (40, 41).On the day of analysis, the plasma aliquots were thawed, vortexed and analyzed for viral load using Roche COBAS® AmpliPrep/COBAS® TaqMan viral assay (42).

### Data analysis

Explanatory variables included age, sex, history and duration of smoking habits, history of use of alcohol, systolic blood pressure, diastolic blood pressure, fasting blood sugar, serum lipid profiles (LDL, HDL, TG, TC), body mass index, waist circumference, history of ARV use, duration on ART, ART regimen-line and class of ARV used. All completed data collection tools as well as all laboratory and radiological results were retrieved and duly entered into Statistical Package for Social Sciences (SPSS) software version 21.0 for storage and analysis (Armonk, NY: IBM Corp).

History of smoking was classified as current smokers: those who have smoked cumulatively in their lifetime 100 cigarettes or more up to a period less than one year preceding the study, previous smokers: those who have smoked cumulatively in their lifetime 100 cigarettes or more but stopped smoking more than one year preceding the study, while non-smokers are those who had smoked cumulatively less than 100 cigarettes in their lifetime or never smoked (43). Systolic blood pressure was classified as normal if <140 mmHg and elevated if ≥140 mmHg, diastolic blood pressure was classified as normal if <90 mmHg and elevated if ≥90 mmHg (44). ARV regimen was classified based on regimen-line into first- or second-line regimen. Regimen was further classified based on its Nucleoside Reverse Transcriptase Inhibitor (NRTI) backbone or the presence of a Non-Nucleoside Reverse Transcriptase Inhibitor (NNRTI), a PI or an integrase inhibitor in the regimen. Duration on ART was categorized as <2 years, 2–4 years and ≥5 years time intervals. Body mass index was classified as <18.5 Kg/m^2^ (underweight), 18.5–24.99 Kg/m^2^ (normal), 25–29.99 Kg/m^2^ (overweight) or >30 Kg/m^2^ (obese) (45). Normal waist/hip ratio was classified as <=0.9 in males, <=0.85 in females. Baseline CD4 count was categorized into <200 mm^3^, 200–349 mm^3^, 350–499 mm^3^ and ≥500 mm^3^. Fasting blood sugar was categorized as <7.0 mmol/L or ≥7.0 mmol/L (46). Fasting lipid profile was classified as Total cholesterol <200 mg/dl or ≥200 mg/dl; LDL < 130 mg/dl or ≥130 mg/dl; HDL < 50 mg/dl or ≥50 mg/dl in females and <40 mg/dl or ≥40 mg/dl in males (47). Viral load was classified as <50 copies/ml (undetectable viral load), 50–199 copies/ml (virally suppressed), 200–999 copies/ml (low level viremia) and ≥1,000 copies/ml (virally unsuppressed). Carotid intima media thickness was categorized as <0.71 mm (non-atherosclerotic) and ≥0.71 mm (subclinical atherosclerotic).

Bivariate analysis using Chi-square test was used to determine factors associated with subclinical atherosclerosis. Factors with a *p*-value of <=0.2 in the bivariate analysis were included in the multivariate model to have a robust mix of predictors to consider. Two multivariable logistic regression models were fitted to explain on the one hand all possible risk factors for atherosclerosis (Model A), while a second model sought to explain the role of HIV infection and ART on atherosclerosis ((Model B). All statistically significant variables from the bivariate analysis were fitted into the multivariable logistic regression model in a stepwise manner to identify all possible risk factors for atherosclerosis (Model A). A second multivariable logistic regression model excluded all the modifiable traditional risk factors of atherosclerosis such as smoking, diabetes mellitus, dyslipidemias, hypertension, and obesity (Model B). A *p*-value <=0.05 was considered statistically significant for all multivariable analysis.

### Ethical considerations

The study protocol was submitted to the human research and ethics committee of UATH for ethical review and approval.

## Results

### Sociodemographic characteristics and associations with subclinical atherosclerosis

Table 1 summarizes the socio-demographic characteristics and habits of study participants. Female participants constituted 178 (64.30%) of the 277 participants enrolled into the study. The mean age of the participants was 39.40 ± 10.70 years with 135 (48.70%) being older than 40 years, and more than half (63.20%) being married. Alcohol use was reported among 40 (14.44%) while majority (90.97%) were non- smokers.

**Table 1:** Socio-demographic characteristics, habits and associations with subclinical atherosclerosis.

Variables		Subclinical atherosclerosis		*p*-value
	*n* (%) or mean SD	Yes (%)	No (%)	
Sex				
Female	178 (64.26)	63 (35.39)	115 (64.61)	<0.001
Age	39.44 (10.71)			
Age group (years)				
20–29	99 (35.74)	3 (5.17)	55 (94.83)	<0.001
30–39	178 (64.26)	27 (32.14)	57 (67.86)	
≥40	39.44 (10.71)	90 (66.67)	45 (33.33)	
Educational status				
None	22 (7.94)	8 (36.36)	14 (63.64)	0.072
Primary	44 (15.88)	24 (54.55)	20 (45.45)	
Secondary	118 (42.60)	42 (35.59)	76 (64.41)	
Tertiary	93 (33.57)	46 (49.46)	47 (50.54)	
Marital status				
Single	66 (23.83)	14 (21.21)	52 (78.79)	<0.001
Married	175 (63.18)	85 (48.57)	90 (51.43)	
Previously married	36 (13.00)	21 (58.33)	15 (41.67)	
Employment status				
Employed	233 (84.12)	105 (45.06)	128 (54.94)	0.178
Unemployed	44 (15.88)	15 (34.09)	29 (65.91)	
Alcohol consumption				
Yes	40 (14.44)	11 (27.50)	29 (72.50)	0.029
Smoking status				
Current smokers	12 (4.33)	6 (50.00)	6 (50.00)	0.132
Previous smokers	13 (4.69)	9 (69.23)	4 (30.77)	
Non-smokers	252 (90.97)	105 (41.67)	147 (58.33)	
Overall	**277 (100)**	**120 (43.32)**	**157 (56.68)**	

SD, standard deviation.

A total of 120 (43.30%) study participants 95% CI: (37.45–49.19) had subclinical atherosclerosis. Subclinical atherosclerosis was more prevalent in males than females (57.60% vs. 35.40%; *p* < 0.001). Prevalence of subclinical atherosclerosis increased with age, *p* < 0.001. Prevalence of subclinical atherosclerosis was higher among participants who reported no alcohol use than those who reported alcohol use (46.0% vs. 27.50%; *p* = 0.03).

### Clinical characteristics and associations with subclinical atherosclerosis

Table 2 shows clinical characteristics of study participants at enrollment. 45.10% of the study participants were over-weight or obese. About 11.19% had systolic blood pressure >140 mm Hg and diastolic blood pressure >90 mm Hg (15.16%). 32.85%, 41.52% and 30% had total cholesterol, low density lipoprotein and triglycerides of >200 mg/dl, >130 mg/dl and >150 mg/dl respectively at enrollment.

**Table 2:** Clinical parameters and associations with subclinical atherosclerosis.

Variables		Subclinical atherosclerosis		*p*-value
	*n* (%)	Yes (%)	No (%)	
BMI (kg/m^2^)				
Under-weight	14 (5.05)	4 (28.57)	10 (71.43)	0.325
Normal	138 (49.82)	55 (39.86)	83 (60.14)	
Over-weight	75 (27.08)	37 (49.33)	38 (50.67)	
Obese	50 (18.05)	24 (48.00)	26 (52.00)	
Systolic BP (mmHg)				
<140	246 (88.81)	98 (39.84)	148 (60.16)	0.001
≥140	31 (11.19)	22 (70.97)	9 (29.03)	
Diastolic BP (mmHg)				
<90	235 (84.84)	97 (41.28)	138 (58.72)	0.104
≥90	42 (15.16)	23 (54.76)	19 (45.24)	
Fasting blood sugar (mg/dl)				
<7	271 (97.83)	116 (42.80)	155 (57.20)	0.408
≥7	6 (2.17)	4 (66.67)	2 (33.33)	
Total cholesterol (mg/dl)				
<200	186 (67.15)	56 (30.11)	130 (69.89)	<0.001
≥200	91 (32.85)	64 (70.33)	27 (29.67)	
Low density lipoprotein (mg/dl)				
<130	162 (58.48)	56 (34.57)	106 (65.43)	<0.001
≥130	115 (41.52)	64 (55.65)	51 (44.35)	
High density lipoprotein				
Normal	12 (4.33)	6 (50.00)	6 (50.00)	0.768
Abnormal	265 (95.67)	114 (43.02)	151 (56.98)	
Waist/hip circumference ratio				
Normal	216 (77.98)	92 (42.59)	124 (57.41)	0.645
Abnormal	61 (22.02)	28 (45.90)	33 (54.10)	
Triglycerides (mg/dl)				
<150	193 (69.68)	71 (36.79)	122 (63.21)	0.001
≥150	84 (30.32)	49 (58.33)	35 (41.67)	

Prevalence of subclinical atherosclerosis was higher among study participants with systolic blood pressure ≥140 mm Hg than those with systolic blood pressure <140 mm Hg (70.97% vs. 39.84%; *p* < 0.001). Subclinical atherosclerosis was more prevalent in study participants with total cholesterol ≥200 mg/dl than those with total cholesterol <200 mg/dl (70.30% vs. 30.10%; *p* < 0.001). Prevalence of subclinical atherosclerosis was higher among study participants with low density lipoprotein *>130 *mg/dl than those with low density lipoprotein <130 mg/dl (55.70% vs. 34.60%; *p* < 0.001). Subclinical atherosclerosis was more prevalent in study participants with triglycerides ≥150 mg/dl than those with triglycerides <150 mg/dl (58.33% vs. 36.79%; *p* = 0.001). Body mass index, diastolic blood pressure, fasting blood sugar, high density lipoprotein, and waist-hip circumference ratio were not associated with subclinical atherosclerosis.

### HIV-related variables and associations with subclinical atherosclerosis

Table 3 highlights HIV-related characteristics of study participants. Of the 277 study participants, 101(66.90%) were on first-line regimen, 254 (91.70%) were in WHO stage I at study enrollment. More than half (54.51%) were ART experienced. Of the 151 ART experienced, 108 (71.50%) have been on ART for ≥5 years and 114 (75.50%) had viral load <50 copies/ml at study enrollment.

**Table 3:** HIV-related variables and associations with subclinical atherosclerosis.

Variables		Subclinical atherosclerosis		*p*-value
	*n* (%) or median (IQR)	Yes (%)	No (%)	
ART status				
ART Naïve	126 (45.49)	26 (20.63)	100 (79.37)	<0.001
ART Experienced	151 (54.51)	94 (62.25)	57 (37.75)	
Median time on ART (IQR) (years)^a^	2.31 (0–10.13)			
Time on ART (years)^a^				
<2 years	9 (5.96)	2 (22.22)	7 (77.78)	<0.001
2–4 years	34 (22.52)	14 (41.18)	20 (58.82)	
≥5 years	108 (71.52) 238 (110- 395)	78 (72.22)	30 (27.78)	
Baseline CD4 (cells/mL)				
<200	107 (38.63)	45 (42.06)	62 (57.94)	0.262
200–349	84 (30.32)	33 (39.29)	51 (60.71)	
350–499	42 (15.16)	24 (57.14)	18 (42.86)	
≥500	44 (15.88)	18 (40.91)	26 (59.09)	
WHO staging at ART initiation				
I	229 (82.67)	96 (41.92)	133 (58.08)	0.116
II	45 (16.25)	24 (53.33)	21 (46.67)	
III/IV	3 (1.08)	0 (0.00)	3 (100.00)	
WHO staging at study enrollment				
I	254 (91.70)	111 (43.70)	143 (56.30)	0.312
II	20 (7.22)	9 (45.00)	11 (55.0)	
III/IV	3 (1.08)	0 (0.00)	3 (100.00)	
Current regimen line				
First- line regimen	101 (66.89)	64 (63.37)	37 (36.63)	0.688
Second-line regimen	50 (33.11)	30 (60.00)	20 (40.00)	
NRTI backbone				
ABC	9 (3.25)	5 (55.56)	4 (44.44)	0.191
TDF	254 (91.70)	106 (41.73)	148 (58.27)	
AZT	14 (5.05)	9 (64.29)	5 (35.71)	
Other drug classes				
NNRTI	17 (6.14)	8 (47.06)	9 (52.94)	0.017
PI	50 (18.05)	30 (60.00)	20 (40.00)	
Integrase inhibitors	210 (75.81)	81 (38.57)	129 (61.43)	
Viral load (copies/ml)^a^				
<50	114 (75.5)	75 (65.79)	39 (34.21)	0.131
50–199	16 (10.60)	7 (43.75)	9 (56.25)	
200–999	6 (3.97)	5 (83.33)	1 (16.67)	
≥1000	15 (9.93)	7 (46.67)	8 (53.33)	

IQR, Interquartile range.

^a^ For ART experienced only.

ART status was associated with subclinical atherosclerosis with 26 (20.60%) of ART naive and 94 (62.30%) of ART experienced patients having subclinical atherosclerosis (*p* < 0.001). Of the 151 ART experienced patients studied, the prevalence of subclinical atherosclerosis increased with increase in the duration of usage ART with a prevalence of 22.20% in those with <2 years usage, 41.20% in those with 2–4 years usage, and 72.20% with ≥5 years usage: *p* < 0.001). 60% of the patients on PI, 47.06% on NNRTI, 38.57% on Integrase inhibitor had subclinical atherosclerosis (*p* = 0.017). There were no significant associations between subclinical atherosclerosis and baseline CD4, WHO staging at ART initiation, WHO staging at study enrollment, current ART regimen-line, and NRTI backbone or viral load.

### Risk factors for subclinical atherosclerosis

Table 4 shows crude and adjusted odds ratio of risk factors for subclinical atherosclerosis adjusted for all risk factors (Model A). Male participants had four-fold odds of subclinical atherosclerosis compared to females [AOR = 4.33, 95% CI (1.74–10.76)]. The odds of having subclinical atherosclerosis increased as age increased, with those 30–39 years having AOR = 5.95; 95% CI (1.31- 26.96), and those ≥ 40 years with AOR = 19.51; 95% CI (4.30–88.56), reference: 20–29 years]. Participants with total cholesterol ≥200 mg/dl had almost four-fold odds of subclinical atherosclerosis compared to those with total cholesterol <200 mg/dl [AOR = 3.88; 95% CI (1.47–10.25)]. Odds of having subclinical atherosclerosis was also higher among participants on ART for ≥5 years than ART naïve participants [AOR = 9.05; 95% CI (3.16–25.92)]. No significant differences were found with marital status, systolic blood pressure, low density lipoprotein, triglycerides, and type of regimen.

**Table 4:** Crude and adjusted associations of subclinical atherosclerosis and socio-demographics, clinical parameters, and HIV-related characteristics (model A).

Variables	UOR (95% CI)	*p*-value	AOR (95% CI)	*p*-value
Sex				
Female	1.00		1.00	
Male	2.48 (1.50–4.10)	<0.001	4.33 (1.74–10.76)	0.002
Age group (years)				
20–29	1.00		1.00	
30–39	8.68 (2.49–30.28)	0.001	5.95 (1.31– 26.96)	0.021
≥40	36.67 (10.87–123.69)	<0.001	19.51 (4.30–88.56)	<0.001
Educational status				
None	1.00		1.00	
Primary	2.10 (0.73–6.01)	0.167	1.92 (0.39–9.41)	0.423
Secondary	0.97 (0.38–2.49)	0.945	1.39 (0.35–5.58)	0.644
Tertiary	1.71 (0.66–4.47)	0.271	2.43 (0.57–10.41)	0.231
Marital status				
Single	1.00		1.00	
Married	3.51 (1.81–6.79)	<0.001	1.24 (0.48–3.18)	0.661
Previously married	5.20 (2.14–12.63)	<0.001	2.30 (0.68–7.81)	0.180
Employment status				
Employed	1.00		1.00	
Unemployed	0.63 (0.32–1.24)	0.180	2.99 (0.94–9.55)	0.064
Smoking status				
Current smokers	1.00		1.00	
Previous smokers	2.25 (0.45–11.52)	0.330	1.55 (0.15–16.62)	0.716
Non-smokers	0.71 (0.22–2.28)	0.569	0.60 (0.11–3.22)	0.555
BMI				
Under-weight	1.00		1.00	
Normal	1.66 (0.49–5.55)	0.413	0.51 (0.10–2.67)	0.429
Over-weight	2.43(0.70–8.45)	0.161	0.70 (0.12–3.99)	0.688
Obese	2.31(0.64–8.35)	0.202	0.54 (0.09–3.30)	0.507
Systolic BP (mm Hg)				
<140	1.00		1.00	
≥140	3.69 (1.63–8.35)	0.002	1.23 (0.30–5.01)	0.770
Diastolic BP (mm Hg)				
<90	1.00		1.00	
≥90	1.72 (0.89–3.33)	0.107	2.20 (0.63–7.66)	0.214
Total cholesterol (mg/dl)				
<200	1.00		1.000	
≥200	5.50 (3.18–9.52)	<0.001	3.88 (1.47–10.25)	<0.001
Fasting blood sugar (mg/dl)				
<7	1.00		1.00	
≥7	2.67 (0.48–14.84)	0.261	2.39 (0.31–18.22)	0.402
Low density lipoprotein (mg/dl)				
<130	1.00		1.00	
≥ 130	2.34 (1.46–3.88)	0.001	0.82 (0.34–1.99)	0.657
High density lipoprotein				
Normal	1.00		1.00	
Abnormal	0.75 (0.24–2.40)	0.63	0.39 (0.07–2.29)	0.295
WHO clinical stage at study enrollment				
I	1.00		1.00	
II & above	0.83 (0.35–1.98)	0.67	4.19 (1.11–15.92)	0.035
Time on ART (years)				
Naïve	1.00		1.00	
<=1	1.10 (0.22–5.61)	0.91	1.05 (0.13–8.29)	0.966
2–4	2.69 (1.20–6.04)	0.016	2.69 (0.77–9.39)	0.121
≥5	10.00(5.47–18.27)	<0.001	9.05 (3.16–25.92)	<0.001
NRTI-regimen based				
ABC	1.00		1.00	
TDF	0.57 (0.15–2.18)	0.415	0.58 (0.08–4.25)	0.592
AZT	1.44 (0.26–7.96)	0.676	2.01 (0.18–23.11)	0.575
Other regimen-base				
NNRTI	1.00		1.00	
PI	1.74 (0.58–5.27)	0.324	1.06(0.19–5.78)	0.947
Integrase	0.71 (0.26–1.92)	0.502	1.17 (0.27–5.04)	0.836
Triglycerides (mg/dl)				
<150	1.00		1.00	
≥150	1.01 (1.00–1.02)	0.017	1.00 (0.99–1.01)	0.682
Baseline CD4 count				
<200	1.00		1.00	
200–349	0.89 (0.50–1.60)	0.70	1.12(0.47–2.70)	0.793
350–499	1.84(0.89–3.78)	0.099	1.69 (0.60–4.82)	0.324
≥500	0.95(0.49–1.07)	0.897	1.54 (0.49–4.78)	0.458

UOR, unadjusted odds ratio; AOR, adjusted odds ratio.

In model B (Table 5) in which modifiable traditional risk factors of subclinical atherosclerosis were excluded, sex, age, WHO clinical stage at study enrollment, and time on ART were found to be risk factors significantly associated with subclinical atherosclerosis. The odds of having subclinical atherosclerosis increased as age increased, with those 30–39 years having AOR = 6.95; 95% CI (1.69- 28.59), and those ≥40 years with AOR = 20.97; 95% CI (5.01–87.69)]. Male participants had almost four-fold increase in the odds of having subclinical atherosclerosis compared to females [AOR = 3.91, 95% CI (1.82–8.39)]. The odds of having subclinical atherosclerosis increased as duration on ART increased with a duration of 2–4 years associated with AOR = 3.24; 95% CI (1.07–9.80), ≥ 5 years duration associated with AOR = 11.43; 95% CI (4.62–28.29), reference: ART naive]. Type of regimen, baseline CD4 count were not associated with subclinical atherosclerosis (Table 5).

**Table 5:** Crude and adjusted associations of subclinical atherosclerosis and socio-demographics, and HIV-related characteristics (model B).

Variables	UOR (95% CI)	*p*-value	AOR (95% CI)	*p*-value
Sex				
Female	1.00		1.00	
Male	2.48 (1.50–4.10)	<0.001	3.91 (1.82–8.39)	<0.001
Age group (years)				
20–29	1.00		1.00	
30–39	8.68 (2.49–30.28)	0.001	6.95 (1.69–28.59)	0.007
≥40	36.67 (10.87–123.69)	<0.001	20.97 (5.01–87.69)	<0.001
Educational status				
None	1.00		1.00	
Primary	2.10 (0.73–6.01)	0.167	1.43 (0.35–5.91)	0.621
Secondary	0.97 (0.38–2.49)	0.945	1.02 (0.30–3.53)	0.971
Tertiary	1.71 (0.66–4.47)	0.271	2.19 (0.60–8.06)	0.238
Marital status				
Single	1.00		1.00	
Married	3.51 (1.81–6.79)	<0.001	1.23 (0.51–2.92)	0.646
Previously married	5.20 (2.14–12.63)	<0.001	2.67 (0.84–8.48)	0.095
Employment status				
Employed	1.00		1.00	
Unemployed	0.63 (0.32–1.24)	0.180	2.22 (0.76–6.51)	0.145
WHO clinical stage at study enrollment				
I	1.00		1.00	
II & above	0.83 (0.35–1.98)	0.67	3.93 (1.19–13.04)	0.025
Time on ART (years)				
Naïve	1.00		1.00	
<=1	1.10 (0.22–5.61)	0.91	1.10 (0.17–7.20)	0.923
2–4	2.69 (1.20–6.04)	0.016	3.24 (1.07–9.80)	0.037
≥5	10.00(5.47–18.27)	<0.001	11.43 (4.62–28.29)	<0.001
NRTI-regimen based				
ABC	1.00		1.00	
TDF	0.57 (0.15–2.18)	0.415	0.48 (0.09–2.70)	0.408
AZT	1.44 (0.26–7.96)	0.676	1.87 (0.21–16.33)	0.571
Other groups				
NNRTI	1.00		1.00	
PI	1.74 (0.58–5.27)	0.324	0.92 (0.21–4.15)	0.916
Integrase	0.71 (0.26–1.92)	0.502	1.23 (0.33–4.65)	0.756
Baseline CD4 count				
<200	1.00		1.00	
200–349	0.89 (0.50–1.60)	0.699	1.17(0.53–2.60)	0.696
350–499	1.84(0.89–3.78)	0.099	1.98 (0.71–5.56)	0.194
≥500	0.95(0.49–1.07)	0.897	1.78 (0.64–4.96)	0.271

UOR, unadjusted odds ratio; AOR, adjusted odds ratio.

## Discussion

### Prevalence of subclinical atherosclerosis

We found an overall prevalence of subclinical atherosclerosis of 43.32% in our study higher than the prevalence of 18% found in the Uganda study. A plausible explanation for the differences in prevalence of subclinical atherosclerosis would be the lower CIMT threshold of ≥0.71 mm used to determine subclinical atherosclerosis in our study compared to ≥0.78 mm in the Uganda study. However, like the Uganda study we found a higher prevalence of subclinical atherosclerosis in men. A systematic review by Abeysuriya et al. identified variations in mean CIMT among healthy persons by sex, age groups and regions, with higher mean CIMT values among males across all regions. Overall mean CIMT was highest in the WHO AFRO (African) region (0.72 mm) and increased with age, with mean CIMT of 0.59 mm, 0.63 mm, 0.76 mm and 0.83 mm in age groups 40–49 years, 50–59 years, 60–69 years and >70 years respectively (48). Our threshold CIMT value is comparable to the mean CIMT reported for the African region and when we consider age differences in mean CIMT, our reference value is higher than thresholds reported for ages 40–59 years. With a mean age of 39.40 ± 10.70 years among our study participants, thus making 68% (mean ± 1SD) of our study participants below 59 years, it presupposes that adopting age specific CIMT threshold would increase our observed prevalence above 68%.

In the bivariate analysis, subclinical atherosclerosis was associated with exposure to ART with a higher prevalence compared to ART naïve patients. This finding is comparable to findings by Sarfo et al. (49), in Ghana, that found a prevalence of 67.60% in patients on ARVs. We found subclinical atherosclerosis to be associated with duration on ART, with participants who had been on ARVs for more than 5 years having a higher prevalence of subclinical atherosclerosis. Maggi et al. (50), in a European Cohort study found 32.20% of HAART- treated patients to have CIMT >1.00 mm. The mechanism of atherosclerosis in HIV positive patients has been linked to immune activation. Evidence suggests that immune activation still persists with the use of ARVs and contributes to accelerated atherosclerosis (51).

In model A, after adjusting for confounders the only significant risk factors identified from our study for subclinical atherosclerosis included male sex, age of 40 years and above, advancing HIV infection (WHO stage II & above at study enrollment) and duration on ART of five years or more. Male participants had four-fold increase in the risk of having subclinical atherosclerosis compared to females similar to the findings of Albuquerque et al. (52) who reported a three-fold increased risk of subclinical atherosclerosis in HIV patients. Studies have shown that men have twice the risk of having coronary heart disease due to atherosclerosis than women. This sex difference has been attributed to a protective effect of female sex hormones, and a deleterious effect of male sex hormones, upon the cardiovascular system. Although the evidence on the harmful effects of testosterone on the heart is limited (53). We found an increased risk of subclinical atherosclerosis with age, similar to the findings of Ssinabuya et al. (27). Increased age has been identified as an independent risk factor for atherosclerosis even when all other factors are controlled (53). Study participants with total cholesterol of 200 mg/dl and above had almost four-fold increase in the odds of presenting subclinical atherosclerosis compared to those with total cholesterol less 200 mg/dl. We also found the odds of having subclinical atherosclerosis to be higher among study participants who were enrolled into care at WHO clinical stage II and above compared to WHO stage I similar to the findings of Desormais et al., who found higher risk of lower extremity arterial disease (LEAD) amongst patients in WHO stage IV compared to stage I using ankle brachial index measurements as an index of subclinical atherosclerosis (54). Similar findings were also reported by Kamdem et al., amongst patients in WHO stages II and IV (55). We found a higher risk of subclinical atherosclerosis among those who have been on ART for five years or above compared to ART naïve participants. Similar findings were reported by Roozen et al. who found the risk of subclinical atherosclerosis to increase with duration on ART (56). Post et al., in their study among male HIV patients amongst men who have sex with men (MSM) found a slightly higher risk of coronary artery stenosis with increasing duration on ART (57). In model B after excluding the modifiable traditional risk factors of subclinical atherosclerosis (including smoking, body mass index, systolic blood pressure, diastolic blood pressure, fasting blood sugar, total cholesterol LDL cholesterol, HDL cholesterol, and triglycerides) male sex, advancing age, advancing HIV infection (WHO stage II & above at study enrollment) and duration on ART ≥5 years were associated risk factors.. These findings suggest that even in the absence of traditional risk factors, advancing HIV infection as well as duration on ART are significant risk factors for development of subclinical atherosclerosis. Gupta et al. (58), had suggested that HIV infection itself together with ART are stronger predictors of atherosclerotic disease than the traditional cardiac risk markers like age, body mass index, diastolic blood pressure, low HDL and history of smoking. Contrary to findings in some older studies (18, 58–61), we found no significant risk differences for subclinical atherosclerosis with systolic blood pressure and diastolic blood pressure, low density lipoprotein, triglycerides and type of regimen. This further supports the notion that HIV infection and ART are stronger predictors of atherosclerotic disease than the traditional risk factors. A large South African study comparing HIV negative patients with HIV positives demonstrated that even with lower levels of traditional risk factors for atherosclerosis among HIV positive patients, there was increased risk of cardiovascular disease among aging population of PLHIV 30 years and above. This study further demonstrated increased risk of CVD with increased duration on ART in the face of limited risk with increased duration of HIV infection, thus suggesting that this risk is largely due to ART (62).

Our study provides better understanding of the prevalence and predictors of subclinical atherosclerosis in HIV positive patients in our environment to inform prevention and management of CVD in PLHIV. However, our study has a few limitations. We employed a cross-sectional study design. A longitudinal or cohort design would have been more appropriate to firmly establish the temporal effect of HIV or ART on the aetiopathogenesis of subclinical atherosclerosis. Another limitation of our study was the lack of longitudinal data on regimen changes (substitutions and switches) as well as duration on specific regimen which would have enabled us to further profile the cardiotoxic effects of specific ARV molecules in the aetiopathogenesis of subclinical atherosclerosis. Furthermore, a lack of data on date of HIV diagnosis for all patients or longitudinal data on viral load assays limits our ability to explore the role of prolonged viraemia in the aetiopathogenesis of CVD. We adopted a cross-sectional design due to funding limitations. The adopted methodology was however sufficient to answer our study questions. Future studies may consider exploring these outstanding questions.

## Conclusion

We found a high prevalence of subclinical atherosclerosis amongst ART experienced persons in our study. Associated risk factors for subclinical atherosclerosis include male sex, advancing age, hypercholesterolaemia, advancing HIV disease, and duration on ART. Even in the absence of traditional risk factors for CVD, advancing HIV infection and duration on ART are significant risk factors for subclinical atherosclerosis.

Our study findings strengthen the case for cardiovascular risk screening amongst people living with HIV.

## Data Availability

The raw data supporting the conclusions of this article will be made available by the authors, without undue reservation.
